# Strategies in product engineering of mesenchymal stem cell-derived exosomes: unveiling the mechanisms underpinning the promotive effects of mesenchymal stem cell-derived exosomes

**DOI:** 10.3389/fbioe.2024.1363780

**Published:** 2024-05-02

**Authors:** Yudong Jiang, Hanning Lv, Fuguo Shen, Lei Fan, Hongjun Zhang, Yong Huang, Jia Liu, Dong Wang, Haile Pan, Jianhua Yang

**Affiliations:** ^1^ Orthopedics Department, The Second Affiliated Hospital of Harbin Medical University, Harbin, China; ^2^ Orthopedics Department, Longgang District People’s Hospital of Shenzhen and the Second Affiliated Hospital, The Chinese University of Hong Kong, Shenzhen, China; ^3^ Orthopedics Department, The Third Affiliated Hospital of Qiqihar Medical University, Qiqihar, China; ^4^ Central Laboratory, Longgang District People’s Hospital of Shenzhen and the Second Affiliated Hospital, The Chinese University of Hong Kong, Shenzhen, China; ^5^ The Biomechanics Group, Department of Mechanical Engineering, Imperial College London, London, United Kingdom; ^6^ Department of Engineering, Faculty of Environment, Science and Economy, University of Exeter, Exeter, United Kingdom

**Keywords:** engineering, osteoarthritis, cartilage damage, induced mesenchymal stem cells, exosomes, 3D printed stent

## Abstract

Articular cartilage injuries present a significant global challenge, particularly in the aging population. These injuries not only restrict movement due to primary damage but also exacerbate elderly degenerative lesions, leading to secondary cartilage injury and osteoarthritis. Addressing osteoarthritis and cartilage damage involves overcoming several technical challenges in biological treatment. The use of induced mesenchymal stem cells (iMSCs) with functional gene modifications emerges as a solution, providing a more stable and controllable source of Mesenchymal Stem Cells (MSCs) with reduced heterogeneity. Furthermore, In addition, this review encompasses strategies aimed at enhancing exosome efficacy, comprising the cultivation of MSCs in three-dimensional matrices, augmentation of functional constituents within MSC-derived exosomes, and modification of their surface characteristics. Finally, we delve into the mechanisms through which MSC-exosomes, sourced from diverse tissues, thwart osteoarthritis (OA) progression and facilitate cartilage repair. This review lays a foundational framework for engineering iMSC-exosomes treatment of patients suffering from osteoarthritis and articular cartilage injuries, highlighting cutting-edge research and potential therapeutic pathways.

## 1 Introduction

Cartilage damage, a consequence of factors such as exercise, trauma, inflammation, and degeneration, represents the most prevalent joint disease globally and is a principal precursor to osteoarthritis (OA) (Berenbaum and Walker, 2020; Quicke et al., 2022). The limited regenerative and repair capabilities of articular cartilage, a consensus among scholars, pose significant challenges; once damaged, its self-repair ability is notably compromised. This progressive deterioration of articular cartilage, both through damage and disease, leads to OA (Coryell et al., 2021). The pathological foundation of OA includes cell inflammation-mediated chondrocyte hypertrophy, degeneration, apoptosis, extracellular matrix (ECM) degradation, and articular cartilage and subchondral bone reactive hyperplasia. Its clinical manifestations, such as pain, joint swelling, stiffness, and deformity, profoundly impact patients’ quality of life, underscoring the urgency of effective articular cartilage repair strategies before OA onset.

Cartilage, a specialized connective tissue, is distinguished by its avascular, aneural, and alymphatic nature, characterized by a dense ECM and chondrocytes embedded in a matrix rich in collagen fibers and proteoglycans (Vahedi et al., 2021). Its histological and biochemical properties, coupled with the low metabolic and proliferative states of chondrocytes, restrict their hyperplasia and migration to damaged areas. This limitation impedes cartilage tissue’s self-repair and regeneration, failing to halt disease progression (Schenker et al., 2017). Current clinical approaches to osteoarthritis and osteochondral injury encompass conventional medication for early-stage articular cartilage damage, which alleviates swelling and pain but fails to fundamentally address lesion progression. Surgical interventions, including subchondral microfractures, autografts, allogeneic cartilage grafts, and periosteal and perichondrial transplantation, face challenges such as tissue degeneration, donor scarcity, and limited repair scopes. Hence, timely and effective treatment of articular cartilage damage remains crucial in preventing OA development.

Tissue engineering and regenerative medicine have recently gained prominence in addressing articular cartilage damage and osteoarthritis. This field leverages bioengineering technologies to repair and regenerate human tissues and organs, combining materials science, medicine, biology, and engineering theories. Techniques include using biological materials as scaffolds for cell and tissue regeneration, employing pluripotent stem cells, and inducing stem cell differentiation with biological activity signal factors to engineer new tissues (Huang et al., 2012; Stanco et al., 2020; Zhao R. et al., 2021). Stem cell-based therapy primarily utilizes human pluripotent stem cells and multipotent (MSCs for regenerative medicine (Hoang et al., 2022). The pioneering stem cell transplantation was performed by the French oncologist George Mathé in 1958 (Jansen, 2005). Over subsequent decades, MSC-based research and therapy have witnessed significant achievements, attributed to MSCs’ unique properties, such as their ability to evade the immune response, availability from various tissue sources, straightforward isolation, rapid proliferative capacity, and suitability for cryopreservation. Recently, the focus has increasingly shifted towards exosomes derived from MSCs, prompted by concerns over the medical safety and the yet-to-be-fully-ascertained efficacy of MSC-based therapies (Hoang et al., 2022).

Compared to cell-based therapies, exosome application offers numerous advantages, including high physicochemical stability, inherent biocompatibility, minimal toxicity, and reduced immunogenicity (Hade et al., 2022; Nan et al., 2022). Furthermore, exosomes exhibit unique biological functions mirroring those of their parent cells (Zhou A. K. et al., 2023). Notably, exosomes serve as promising drug delivery vehicles, attributed to their prolonged circulation half-life, favorable biodistribution, specific cellular interactions, and the feasibility of direct modification (Kimiz-Gebologlu and Oncel, 2022; Nan et al., 2022). Extracellular vesicles (EVs), particularly exosomes, have been identified as key mediators of MSC paracrine function, playing a vital role in intercellular signaling (Chen et al., 2019). Exosomes, derived from transcytosed endosomes and released into the extracellular fluid, are lipid bilayer membrane-enclosed structures containing various bioactive molecules. They function as intercellular communication pathways, facilitating material exchange and signal transmission between cells (Patil and Rhee, 2019; Skotland et al., 2019). In stem cell biological therapy, exosomes mimic parental stem cell functions while avoiding issues like abnormal differentiation and immune rejection.Futhermore, it is easier to preserve and transport them (Atlasi and Stunnenberg, 2017; Zhou C. et al., 2023). The application of exosomes in OA prevention and treatment has shown promising results, such as their role in the formation of neohyaline cartilagein rat knee cartilage defect models, effectively promoting cartilage matrix synthesis and delaying articular cartilage degeneration (Zhang et al., 2015; Zhu et al., 2017).

In this review, we consolidate the benefits and manufacturing techniques associated with engineering exosomes. Additionally, we delve into recent research that elucidates the mechanisms underpinning the therapeutic effects of MSC-derived exosomes on OA.

## 2 The advantage and challenge of induced mesenchymal stem cells (iMSCs)-exosomes

In comparison to MSCs isolated from homologous adult tissues, iMSCs exhibit a more stable molecular phenotype, enhanced proliferative differentiation capabilities, and superior regenerative repair capacities in animal disease models (Sabapathy and Kumar, 2016; Lee et al., 2023; Gultian et al., 2022). Consequently, iMSCs, surpassing iPSCs in quality, present broader application prospects. They inherit the advantages of iPSCs, including the ability to reprogram diverse adult cells such as those from peripheral blood, skin biopsies, and detached body surface cells (; Dupuis and Oltra, 2021; Wang Q. et al., 2023; Wu et al., 2018; Zhou C. et al., 2023). Given the potentially infinite replicative capacity of iPSCs, the source for iMSCs can be considered similarly inexhaustible. While iPSCs themselves display heterogeneity, a single iPSC clone exhibits low internal heterogeneity (Devito et al., 2019). Thus, deriving iMSCs from single iPSC cells or clonal differentiation minimizes MSC heterogeneity, facilitating the acquisition of functionally stable and defined MSC products, including exosomes. iMSCs can be efficiently prepared on a large scale, yielding stable and controllable final products, including exosomes, suitable for diverse clinical applications downstream (Obara et al., 2016)

Furthermore, the monoclonal stage of iPSCs is conducive to genetic modifications, influencing all downstream iMSC products, including exosomes (Hollmann et al., 2020). This capability opens avenues for developing new generations of biotherapeutic technologies leveraging specific gene functions.The efficacy of exosome-based biotherapies is intricately linked to the source of the parental stem cells. Advances in cell reprogramming technology, particularly in the study of induced pluripotent stem cells (iPSCs), have not only deepened our understanding of stem cell pluripotency and its molecular regulatory mechanisms but have also provided a source of totipotent stem cells capable of extensive proliferation (Gallegos et al., 2013; Aboul-Soud et al., 2021).

However, the research and development of iMSC-exosomes face several technical challenges, including (; Zhang et al., 2023; Wang X. et al., 2023; Chen et al., 2021; Sadeghi et al., 2023; Gurung et al., 2021; Hade et al., 2021; Tang et al., 2023): 1) Identifying the core efficacy components and mechanisms of action of iMSC-exosomes for specific diseases. 2) Enhancing the enrichment and efficacy of specific components within iMSC-140 exosomes. 3) Ensuring mass production, quality stability, and efficient storage and transportation processes for iMSC-exosomes. 4) Optimizing the effective delivery and functionality of iMSC-exosomes in specific tissue contexts.

## 3 Strategies to improve exosome performance

**FIGURE 1:**
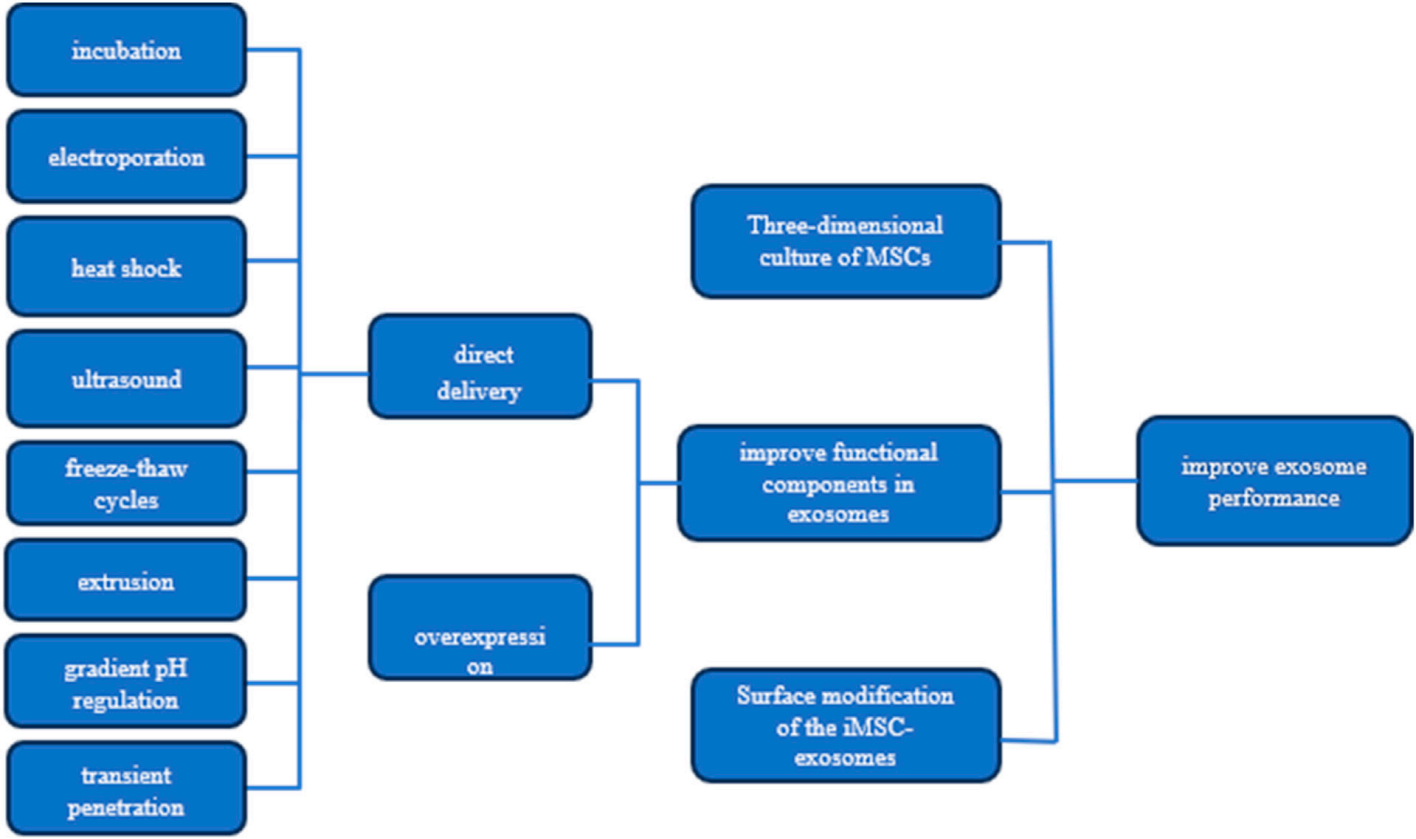
Strategies for Enhancing Exosome Efficacy This figure presents three key strategies for boosting the performance of MSC-derived exosomes: 1) three-dimensional culture of MSCs to enhance exosome production and quality, 2) improving exosomal functional components through targeted pathways, and 3) surface modification of exosomes for better targeting and delivery. Additionally, it illustrates methods to increase the concentration of effective molecules (effectors) within the exosomes.

To augment the yield and quality of exosomes, as well as to enhance their targeting precision, biostability, and therapeutic efficacy, numerous studies have been conducted. These accomplishments are comprehensively summarized in Figure 1.

### 3.1 Three-dimensional culture of MSCs

The conventional method of culturing mesenchymal stem cells (MSCs) involves a two-dimensional (2D) monolayer setup. However, this approach has limitations, particularly in terms of proliferation efficiency, falling short of meeting the clinical demand for high doses of MSCs. Furthermore, the development and functionality of MSCs are significantly influenced by molecular interactions within their environment. Traditional 2D cultures fail to replicate the spatial distribution, nutrient transfer, cell migration, and mechanical stimulation characteristics of a three-dimensional (3D) extracellular matrix (ECM) microenvironment. This discrepancy can lead to variations in gene expression, signaling, and morphology of MSCs from their natural state (Li et al., 2015), potentially impacting the biological function of exosomes.

**FIGURE 2:**
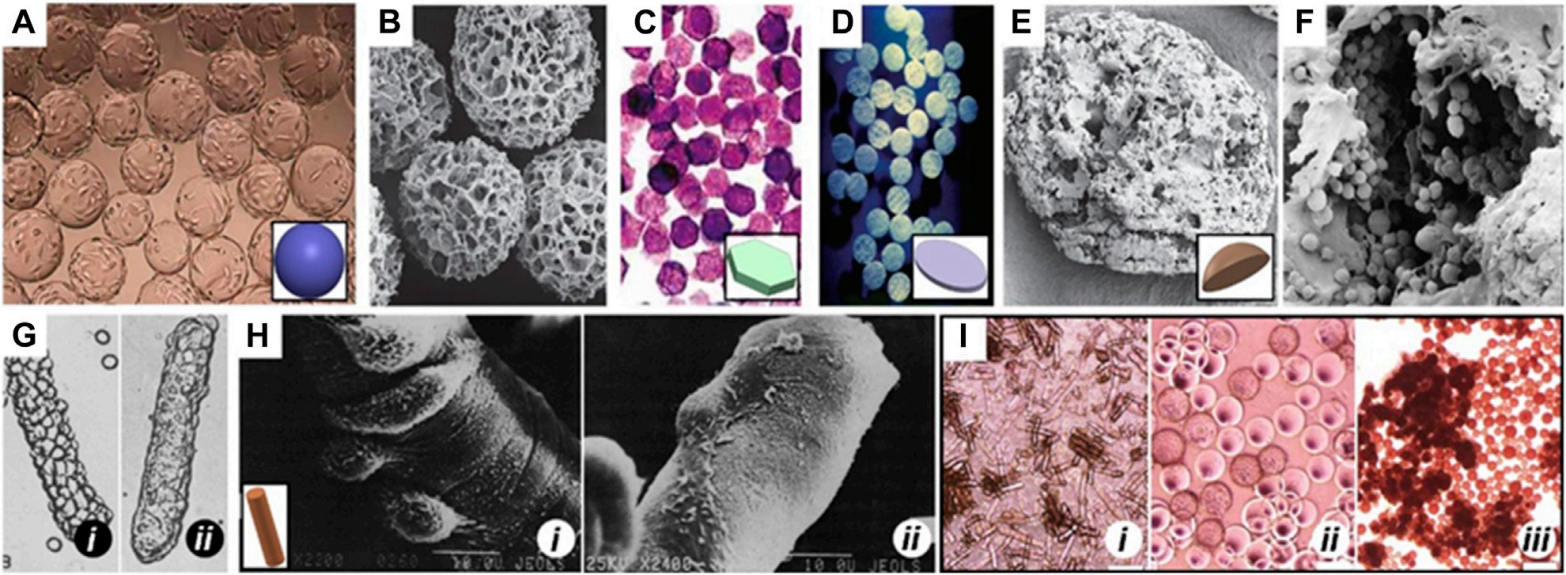
MCs of different shapes Spherical MCs like **(A)** Cytodex 1 and **(B)** Cytopore. **(C)** 2D microhex is an example of hexagon shape carriers produced by Nunc™. **(D)** Fibra-cel^®^ is a product of New Brunswick™ that has a disc shape. **(E)** Lens-shape MCs like Cytoline 1 and Cytoline 2 **(F)** are produced via GE Healthcare. **(G)** and **(H)** shows DE-52 and DE-53 as available cylindrical MCs in the market which are produced by Whatman™. In panel G, i and ii shows attachment of L-929, a corn-type fibroblastic and MDCK, a canine kidney cell line, Epithelial cells, respectively to DE-52. In panel H, i and ii shows attachment of the baby hamster kidney cell line (BHK, Fibroblastic type) and Primary chick embryo fibroblast, respectively to DE-53. **(I)** Morphology of human ESCs on (i) DE-53 (large aggregates), (ii) Cytodex-1 (thin aggregate layers) and (iii) Tosoh 65 PR (compact aggregates). Images adapted from Ref (Niet al., 2020).

Recognizing these limitations, the 3D culture and amplification of MSCs using 3D printed scaffolds have gained widespread acceptance. Microcarriers (MCs), consisting of hydrogel microparticles made from materials like gelatin, hyaluronic acid, collagen, or natural biological macromolecules such as chitosan, sodium alginate, and their derivatives combined with polyethylene glycol, offer a promising solution (Figure 2) (Tavassoli et al., 2018). These MCs present several advantages, including facilitating large-scale cell culture and expansion, mimicking *in vivo* 3D microenvironments, establishing cell-cell and cell-ECM interactions, and promoting gene expression and secretion activities akin to *in vivo* conditions (; Tseng et al., 2016; Shekaran, et al., 2016).

### 3.2 The approaches to improve the functional components in exosomes

The extraction of exosomes typically involves techniques such as overspeed centrifugation, ultrafiltration, immunoaffinity capture, charge-neutralization-based polymer precipitation, size exclusion chromatography, and microfluidic separation (Wu J. et al., 2019). These methods yield exosomes with varying levels of purity, yield, size, surface potential, and inclusion composition, and currently, there is no standardized extraction protocol (Yang et al., 2019). The identification of exosomes is predominantly based on their morphological characteristics under electron microscope, particle size specific protein markers (Zhao X. et al., 2021). However, the efficacy of these identification methods can be influenced by the quality of the kits used and other external factors (Momen-Heravi F. 2017).

To enhance tissue regeneration efficiency, further enrichment of functional components in exosomes is crucial (Sun et al., 2021). The enrichment of exosomes with functional components is primarily achieved through either the overexpression of these components in parental cells or direct delivery to the exosomes (Pegtel and Gould, 2019). The former approach, involving genetic expression of functional components by parental cells and their subsequent secretion into exosomes, faces challenges in specifically regulating functional components and often results in low yields of exosomes enriched with targeted components (Sun et al., 2021). In contrast, physical and chemical methods such as incubation, electroporation, heat shock, ultrasound, freeze-thaw cycles, extrusion, gradient pH regulation, and transient penetration of phospholipid bilayers have proven more efficient and feasible (Colombo et al., 2014; Kalluri and LeBleu, 2020; Mondal et al., 2023). These methods enhance the interaction of target functional components with the exosomes’ surface.

### 3.3 Surface modification of the iMSC-exosomes

The lipid bilayers of exosomes play a crucial role in shielding internal functional components from external environmental damage. However, their thermodynamic instability during storage makes them susceptible to alterations in the medium, temperature, time, and physical and chemical properties. Such changes can result in the loss of internal functional components, formation of multilayer vesicle structures, and aggregation. Repeated freeze-thaw cycles can also alter the lipid bilayer’s marker characteristics and composition (Maroto et al., 2017).

When administered subcutaneously, intravenously, or intraperitoneally, exosomes are known to accumulate in organs like the liver, spleen, lungs, and gastrointestinal tract within 2 h and are rapidly cleared by macrophages (Takahashi et al., 2013). The incorporation of exosomes into hydrogels for sustained release can mitigate the low tissue retention observed with direct injections. However, this passive diffusion release mechanism is difficult to control due to the influence of hydrogel porosity and degradation rate. Moreover, the negative charge on both exosome and cell membranes creates electrostatic barriers that reduce the bio-utilization efficiency of exosomes (Feng et al., 2021).

Surface modification of exosomes can enhance their physicochemical properties. Sushil et al. utilized cholesterol-modified DNA strands for complementary pairing and embedding a polymer onto the exosome membrane surface, improving stability and therapeutic efficacy without altering *in vivo* distribution or specific targeting. However, this method’s chemical synthesis complexity and the temperature-sensitive annealing of DNA chain segments may impact exosome activity. In contrast, Feng et al. (2021) demonstrated the incubation of exosomes with polylysine (ε PL)-PEG-distearoyl phosphatidylethanolamine (DSPE) (PPD), integrating the DSPE segment into the exosome membrane while reversing the ε PL segment from electronegative to electropositive. This modification increased exosome uptake, penetration, and retention in cartilage, thereby enhancing OA treatment effects (Lathwal et al., 2021). While this approach is simpler and does not affect the structure and content of exosomes, it results in increased exosome size and reduced cartilage absorption rate.

## 4 Molecular and cellular mechanisms of MSCs-exosomes in the treatment of OA and articular cartilage

**FIGURE 3:**
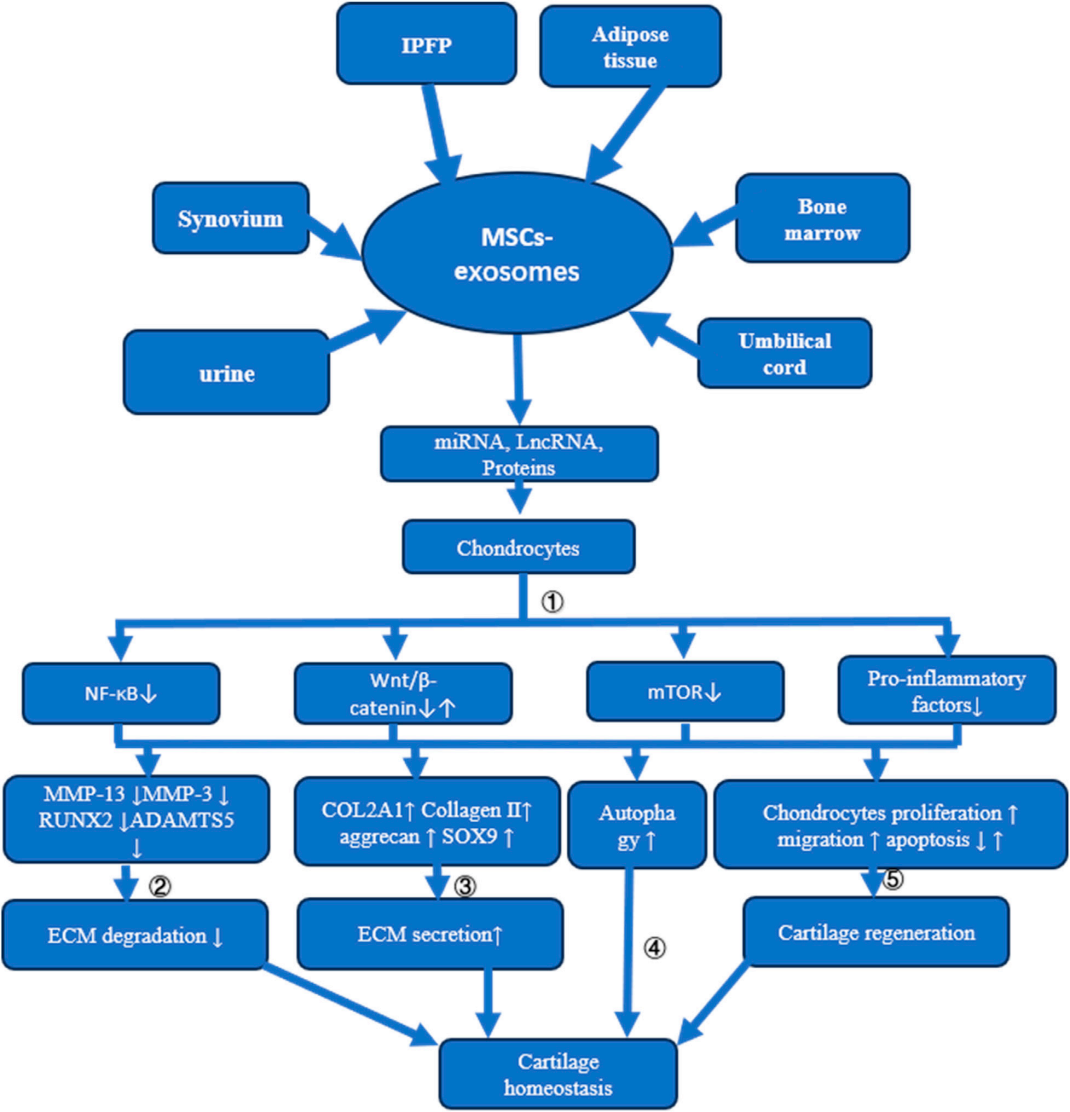
Mechanisms of MSC-Exosome Action in Cartilage Homeostasis and Osteoarthritis Treatment This figure illustrates how MSC-exosomes, derived from different tissues, influence cartilage homeostasis and address osteoarthritis through multiple mechanisms: ① Delivery of miRNA, LncRNA, or proteins to chondrocytes suppresses NF-κB, mTOR, and pro-inflammatory factor release. While some MSC-exosomes may activate the Wnt/β-catenin signaling pathway, overexpression of certain miRNAs redirects these exosomes to inhibit this pathway; ②The suppression of MMP13, MMP3, RUNX2, and ADAMTS5 expression through various signaling pathways prevents ECM degradation; ③ Enhancement of COL2A1, Collagen II, aggrecan, and SOX9 expression via diverse signaling routes increases ECM synthesis; ④ Cartilage homeostasis is maintained by activating autophagy through multiple pathways; ⑤ Cartilage regeneration is promoted by stimulating chondrocyte proliferation and migration and reducing apoptosis via varied signaling mechanisms.

Given the vast array of sources for MSCs, including bone marrow, synovium, umbilical cord, urine, infrapatellar fat pad, and adipose tissues, exosomes derived from these cells hold considerable potential for OA therapy. The exploration of the mechanisms through which MSC-exosomes prevent OA represents a critical pathway towards their clinical application. MSC-derived exosomes contribute to cartilage repair and OA prevention through various mechanisms, such as inhibiting ECM degradation and chondrocyte apoptosis, promoting ECM secretion and autophagy, and enhancing chondrocyte proliferation and migration (refer to Figure 3 and Table 1).

**TABLE 1:** Summary sheet about the sources of MSCs, experimental animal model, methods to enrich exosomes, and key activator molecules in studies about the mechanism of MSCs-exosomes to treat OA.

Exosome source	Model	Separation method	Cargo	Target gene	References
AT-MSCs	Rat	Ultracentrifugation	miR-338-3p	RUNX2	Li et al. (2022a)
BM-MSCs	Mice	Ultracentrifugation	miR-125a-5p	E2F2	Xia et al. (2021)
BM-MSCs	Mice	Ultracentrifugation	miR -92a-3p	WNT5A	Mao et al. (2018)
BM-MSCs		ExoQuick Isolation Kit	miR-320c	MMP-13	Sun et al. (2019)
BM-MSCs	Mouse	Ultracentrifugation	KLF3-AS1	MiR206	Liu et al. (2018)
BM-MSCs	Mice	Ultracentrifugation	miR-136-5p	ELF3	Chen et al. (2020)
BM-MSCs	Mice	Ultracentrifugation	miR -3960	PHLDA2	Ye et al. (2022)
BM-MSCs	Rat	Ultracentrifugation	miR-361-5p	DDX20	Tao et al. (2021)
IPFP-MSCs	Rabbit and rat	ExoQuick™(EQ) reagent kit (SBI) and ultrafiltration	miR-100-5p	mTOR	Wu J. et al. (2019)
S-MSCs	Mouse	Ultracentrifugation	miR-129-5p	HMGB1	Qiu et al. (2021)
S-MSCs	Rat		miR-140-5P, wnt5a,wnt5b	YAP,RalA	Tao et al. (2017)
S-MSCs	Mouse		miR-155-5p	Runx2	Wang et al. (2021)
UC-MSCs	Rat	Ultracentrifugation	miR-1208	METTL3	Zhou et al. (2022)
UC-MSCs	SD rat	Precipitation	LncRNA H19		Yan et al. (2021)
Urine MSCs		Gradient centrifugation	miR-26a-5p	PTEN	Wan et al. (2022)
Urine MSCs	Rat	Ultracentrifugation	miR-140-5p	VEGFA	Liu et al. (2022)

Abbreviation: OA, osteoarthritis; MSC, mesenchymal stem cells; AT-MSCs, adipose tissue derived MSCs; BM-MSCs, bone marrow derived MSCs; IPFP-MSCs, infrapatellar fat pad derived MSCs; S-MSCs, synovial MSCs; UC-MSCs, umbilical cord derived MSCs; PHLDA2, Pleckstrin homology-like domain, family A, member 2; MMP-13, matrix metallopeptidase-13; ELF3, early flowering 3; DDX20, DEAD-box, helicase 20; MTOR, mechanistic target of rapamycin; HMGB1, high mobility group box-1; YAP, Yes-associated protein; RUNX2,runt-related transcription factor 2; METTL3,methyltransferase-like 3; PTEN, phosphatase and tensin homolog; VEGFA, vascular endothelial growth factor A.

### 4.1 Exosomes derived from bone marrow MSCs

Exosomes derived from bone marrow MSCs play a crucial role in mitigating chondrocyte injury in OA induced by IL-1β, with miR-3960 emerging as a principal effector. In chondrocytes, the increase in PHLDA2 expression activates the Wnt/β-catenin pathway through the upregulation of SDC1 expression, leading to the degradation of the cartilage extracellular matrix due to elevated levels of MMP-13 and ADAMTS5, alongside reduced levels of collagen II and aggrecan. However, miR-3960, enriched in the exosomes secreted by MSCs, can be delivered to chondrocyte cells, subsequently degrading PHLDA2 mRNA. Moreover, the expression profiles of miR-3960, PHLDA2, SDC1, and β-catenin in cartilage tissues from OA patients align with findings from their prospective study (Ye et al., 2022).Xia Qingqing et al. identified a negative correlation between miR-125a-5p and E2F2 in the cartilage of traumatic osteoarthritis patients and in a corresponding mouse model. The exosomes derived from bone marrow MSCs, which are taken up by chondrocytes in mice, contain miR-125a-5p that suppresses E2F2 expression. This downregulation leads to decreased MMP-13 levels and increased levels of collagen II, aggrecan, and SOX9, thereby facilitating chondrocyte migration and alleviating extracellular matrix degradation (Xia et al., 2021).Through miRNA microarray analysis, Mao Guping et al. found that miR-92a-3p levels in MSC exosomes were upregulated following chondrogenic induction in bone marrow MSCs, but downregulated in OA chondrocyte-secreted exosomes compared to normal cartilage. MiR-92a-3p suppresses MMP-13 and RUNX2 expression while enhancing aggrecan, COL2A1, and SOX9 expression, thereby inhibiting cartilage degradation—a process regulated by reduced WNT5A expression in chondrocytes (Mao et al., 2018).Sun Hao et al. demonstrated that miR-320c, enriched in exosomes secreted by bone marrow MSCs undergoing chondrogenic differentiation, promotes osteoarthritis chondrocyte proliferation, enhances SOX9 expression, and inhibits MMP-13 expression. This contributes to chondrogenesis induction and inflammation suppression in chondrocytes (Sun et al., 2019).Chen Xue et al. observed higher ELF3 levels and lower miR-136-5p levels in traumatic OA cartilage tissues compared to healthy cartilage. Exosomes secreted by bone marrow MSCs, rich in miR-136-5p, are internalized by chondrocytes, leading to downregulated ELF3 expression, which in turn alleviates cartilage degeneration and enhances chondrocyte migration through the upregulation of collagen II, aggrecan, and SOX9, and the downregulation of MMP-13 expression (Chen et al., 2020).DDX20, identified as a key regulator in chondrocyte damage induced by IL-1β, activates the NF-κB signaling pathway, promoting chondrocyte damage through increased expression of MMP-3 and MMP-13. Exosomes derived from bone marrow MSCs can counteract this effect by downregulating DDX20 levels, thanks to the enrichment of miR-361-5p within them (Tao et al., 2021).Liu Yubao et al. demonstrated that in a mouse OA model, MSC-exosomes promoted chondrocyte proliferation, inhibited apoptosis, and maintained cartilage homeostasis. This was achieved through the upregulation of COL2A1 and aggrecan and the downregulation of MMP-13 and RUNX2, mediated by the lncRNA-KLF3-AS1/miR-206/GIT1 axis (Liu et al., 2018).

### 4.2 Exosomes derived from synovial MSCs

Exosomes derived from synovial MSCs have demonstrated a capacity to foster chondrocyte regeneration, although they did not show benefits for ECM synthesis. These exosomes aid in OA prevention by encouraging chondrocyte proliferation and migration and by reducing apoptosis, yet they do not affect ECM secretion. However, exosomes overexpressing miR-155-5p were found to enhance ECM secretion in OA chondrocytes through the degradation of Runx2 (Wang et al., 2021). In a similar vein, Tao Shicong et al. reported that exosomes from synovial MSCs could activate YAP through the delivery of wnt5a and wnt5b, key activators of the Wnt signaling pathway. This activation leads to increased chondrocyte proliferation and migration, albeit at the expense of ECM secretion. Nonetheless, this limitation could be addressed by miR-140-5p, which elevates the levels of SOX9, aggrecan, and collagen II by inhibiting RalA (Tao et al., 2017). Further, Qiu Min et al. observed a reduced expression of miR-129-5p in the cartilage tissues of OA patients and in chondrocytes induced by IL-1β, while HMGB1 was more highly expressed compared to healthy tissue. Exosomes from human synovial MSCs, rich in miR-129-5p, were able to elevate its levels in chondrocytes, subsequently downregulating HMGB1. Consequently, there was a reduction in the inflammatory response and apoptosis of chondrocytes (Qiu et al., 2021).

### 4.3 Exosomes derived from umbilical cord MSCs

Yan Litao et al. demonstrated that mechanical stimulation during cell culture could amplify the secretion of exosomes by umbilical cord mesenchymal stem cells. These exosomes proved advantageous for repairing cartilage defects, a benefit attributed to the encapsulation of LncRNA H19 (Yan et al., 2020). Zhou Hao et al. further revealed that exosomes derived from umbilical cord mesenchymal stem cells exerted a protective effect on OA, as evidenced in a mouse OA model created by surgical destabilization of the medial meniscus. The protective mechanism was attributed to exosomal miR-1208, which reduced the m6A modification level of NLRP3 mRNA by targeting METTL3 mRNA, leading to a decrease in pro-inflammatory factor levels. Consequently, chondrocyte regeneration was enhanced due to increased proliferation and migration and reduced apoptosis. Additionally, ECM degradation was curtailed through the upregulation of COL2A1 and aggrecan and the downregulation of ADAMTS5 and MMP-13 (Zhou et al., 2022).

### 4.4 Exosomes derived from urine MSCs

Exosomes secreted by urine-derived stem cells cultured under hypoxic conditions were found to be enriched in miR-26a-5p, which effectively inhibits PTEN expression in chondrocytes, thereby promoting their proliferation and migration (Wan et al., 2022). Moreover, exosomes from human urine-derived stem cells were shown to enhance the proliferation and migration of chondrocytes induced by IL-1β, while concurrently inhibiting apoptosis, albeit with a reduction in ECM secretion. Intriguingly, when overexpressed with miR-140-5p, these exosomes facilitated the upregulation of collagen II and aggrecan. Mechanistically, miR-140-5p was found to decrease VEGFA levels in cartilage and synovial fluid samples obtained from patients with knee osteoarthritis or from a rat model of the disease (Liu et al., 2022).

### 4.5 Exosomes derived from remaining MSCs

*In vitro* studies reveal that exosomes derived from adipose stem cells effectively downregulate the expression of prostaglandin E2, IL-6, IL-1β, and TNF-α in the murine chondroprogenitor cell line ADTC5. The encapsulated miR-338-3p within these exosomes is delivered to interleukin-1β-induced ADTC5 cells, promoting the degradation of RUNX2 mRNA. Consequently, this results in the inhibition of cell degradation and apoptosis, alongside the promotion of cell proliferation (Li et al., 2022).Wu Jiangyi et al. demonstrated that exosomes secreted by infrapatellar fat pad MSCs can maintain cartilage homeostasis by enhancing autophagy levels, attributed to the inhibition of mTOR. The encapsulated miR-100-5p within these exosomes can bind to the 3′-untranslated region (3′UTR) of mTOR, promoting its degradation. The exosomes ameliorated OA severity *in vivo* and inhibited cell apoptosis, enhanced matrix synthesis, and reduced the expression of catabolic factors *in vitro*. Given the relative ease of harvesting infrapatellar fat pad MSCs from OA patients, this study may guide future clinical therapies for OA (Wu X. et al., 2019).

## 5 Discussion and perspectives

This review underscores the transformative potential ofiMSC-exosomes in treating OA and repairing articular cartilage. The advanced bioengineering of iMSCs, rooted in iPSC technology, presents a paradigm shift in regenerative medicine. Their enhanced stability, proliferative capabilities, and regenerative repair capacities herald a new era in tissue engineering. However, the journey from laboratory to clinic is fraught with challenges, notably in identifying and enhancing key efficacy components of iMSC-exosomes, ensuring their mass production and stability, and optimizing delivery mechanisms.

The shift from two-dimensional to three-dimensional culture systems using MCsmarks a significant advancement in MSC culture, more accurately mimicking *in vivo* conditions and enhancing exosome functionality. The challenges, such as inefficient cell apposition and particle residue, represent crucial areas for future research. Additionally, the refinement of exosome extraction and enrichment techniques, alongside innovative strategies for surface modification, could exponentially enhance their therapeutic potential.

The intricate molecular and cellular mechanisms by which MSC-exosomes influence cartilage repair and OA treatment, including modulation of inflammatory responses and promotion of chondrocyte proliferation and migration and ECM secretion, inhibition of apoptosis and ECM degradation, are at the forefront of current research.

While certain studies have highlighted the adverse effects of MSC-derived exosomes on the ECM, modifications such as overexpressing specific effectors in MSC-exosomes have demonstrated the potential to counteract these drawbacks. Specifically, the Wnt signaling pathway, which can be activated by certain MSC-exosomes, may be subdued through the overexpression of particular effectors (Tao et al., 2017; Wang et al., 2021; Liu et al., 2022). Consequently, engineered MSC-exosomes, enriched with tailored effector molecules, present a more promising approach for OA prevention compared to their unmodified counterparts. This suggests that strategic modifications can enhance the therapeutic utility of MSC-exosomes, making them a more suitable option for OA intervention. Translating these mechanisms into effective clinical interventions remains a pivotal challenge. Future research must focus on elucidating and harnessing these mechanisms more effectively, potentially through targeted gene editing and advanced bioengineering techniques.

Looking forward, the field must prioritize the development of standardized protocols for iMSC-exosome production and application, addressing issues of heterogeneity and scalability. Clinical trials are imperative to translate these cellular and molecular insights into real-world therapies. Furthermore, exploring the immunomodulatory potential of MSC-exosomes could open new avenues for treating inflammatory aspects of OA.

The potential of iMSC-exosomes in OA treatment and cartilage repair is immense. The intersection of cellular biology, molecular engineering, and bioinformatics stands to revolutionize our approach to regenerative medicine. As we advance, a collaborative, interdisciplinary effort encompassing researchers, clinicians, and regulatory bodies will be essential in overcoming existing barriers and realizing the full therapeutic potential of iMSC-exosomes.

## 6 Conclusion

To enhance the biological treatment of OA and articular cartilage injury, several technical challenges must be addressed. Here we proposed solutions include using functionally genetically modified iMSCs combined with 3D printed scaffolds for 3D culture, enriching functional miRNA, and modifying the membrane surface of iMSC-exosomes to augment their function. We also summarized the mechanisms of iMSC-exosomes on chondrocyte proliferation, apoptosis, autophagy, ECM synthesis, and inflammatory repair at molecular and subcellular levels. The content that discussed in this review offers insights for treating OA and articular cartilage damage.

## Abbreviations

MSCs, mesenchymal stem cells; miRNA, microRNA; lncRNA, long non-coding RNA; NF-κB, nuclear factor kappa B; mTOR, mechanistic target of rapamycin; MMP-13, matrix metallopeptidase-13; MMP-3, matrix metalloproteinase-3; RUNX2,runt-related transcription factor 2; ADAMTS5, a disintegrin and metalloproteinase with thrombospondin motifs 5; COL2A1, collagen type II alpha 1; SOX9, (sex determining region Y)-box 9; ECM, extracellular matrix.
